# Extraordinary response to crizotinib in a woman with squamous cell lung cancer after two courses of failed chemotherapy

**DOI:** 10.1186/1471-2466-14-83

**Published:** 2014-05-15

**Authors:** Qiushi Wang, Yong He, Xin Yang, Yubo Wang, Hualiang Xiao

**Affiliations:** 1 Department of Pathology, Daping Hospital and Research Institute of Surgery, Third Military Medical University, Chongqing 400042, China; 2 Department of Respiration, Daping Hospital and Research Institute of Surgery, Third Military Medical University, Chongqing 400042, China

**Keywords:** *ALK*, Crizotinib, Squamous cell lung cancer, Chemotherapy

## Abstract

**Background:**

The discovery of the fusion gene echinodermmicro tubule associated proteinlike 4-anaplastic lymphoma kinase, EML4-ALK, in patients with non-small-cell lung cancer has led to the remarkable development of anaplastic lymphoma kinase inhibitors, such as crizotinib. Consequently, the clinical outcomes of these patients have improved dramatically. Herein, we report the case of a woman with *ALK* gene translocation-squamous cell lung cancer who experienced a remarkable tumor response to crizotinib after two courses of failed chemotherapy.

**Case presentation:**

A 55-year-old Chinese woman was diagnosed with cervical lymph node metastatic squamous carcinoma. Chest computed tomography scan showed the primary tumor in the lower lobe of the right lung. The patient had received two successive courses of first-line chemotherapy without tumor response. Tumor cells were negative for wild-type of epidermal growth factor receptor/K-RAS variants; thus, she was not eligible for tyrosine kinase inhibitor therapy. Unfortunately, increased levels of interleukin-6 and carcinoembryonic antigen, and computed tomography scan results indicated cancer progression. Once crizotinib was approved by the China Food and Drug Administration and the *ALK* gene translocation was identified in tumor cells by fluorescent in situ hybridization, the patient commenced treatment with crizotinib. Remarkably, the tumor response to crizotinib was classified as partial response after only 26 days of treatment commencement. The partial response status has been maintained to date (23 weeks).

**Conclusion:**

Considering this remarkable response to crizotinib, we can safely conclude that patients with squamous cell lung cancer should have the option of undergoing *ALK* testing to determine if there is indication for crizotinib treatment even after they have failed chemotherapy.

## Background

Treatment of EML4-ALK fusion-positive non-small-cell lung cancer (NSCLC) with the anaplastic lymphoma kinase (ALK)-targeted agent, crizotinib, offers significant improvement of clinical outcomes [1]. Herein, we report the successful case of a patient with squamous cell lung cancer and ALK gene translocation that experienced a remarkable response to crizotinib treatment after two courses of failed chemotherapy.

## Case presentation

**Figure 1:**
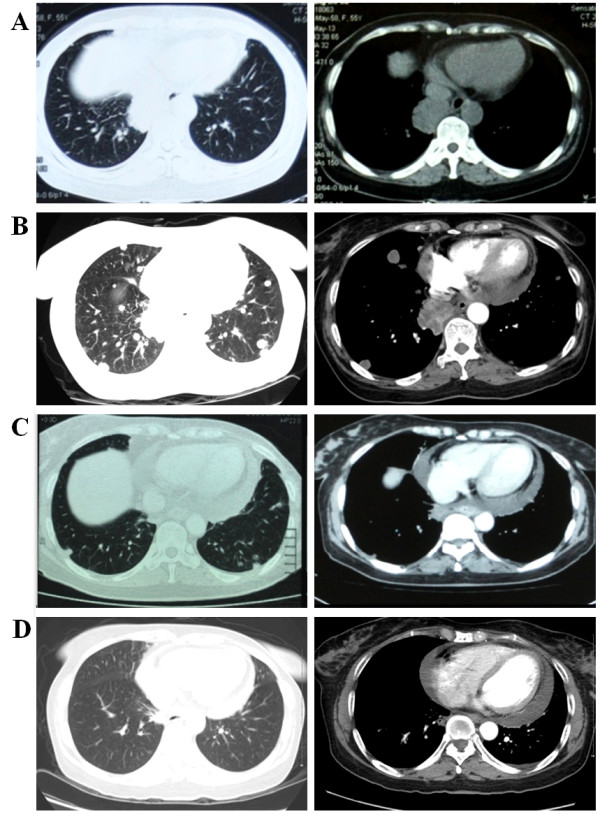
**Chest computed tomography (CT) scans.** Before the first chemotherapy treatment (May 2013) **(A)**. After the second course of chemotherapy **(B)**. After 26 days **(C)** and 11 weeks **(D)** of crizotinib treatment.

A 55-year-old woman presented in May 2013 with cough, sputum, and annihilation after activities of daily living, for more than 20 days. She had no history of smoking or alcoholism, but had undergone total hysterectomy because of hysteromyoma in 2010. She was diagnosed with hypertension three years earlier. On physical examination, an enlarged right cervical lymph node was palpated. Chest computed tomography (CT) scan (Figure 1A) indicated the presence of a mass in the lower lobe of the right lung and mediastinal lymph node enlargement. The patient was then accepted and treated by the Department of Respiration for lung cancer stage IV with cervical lymph node metastasis (T4N3M1).

**Figure 2:**
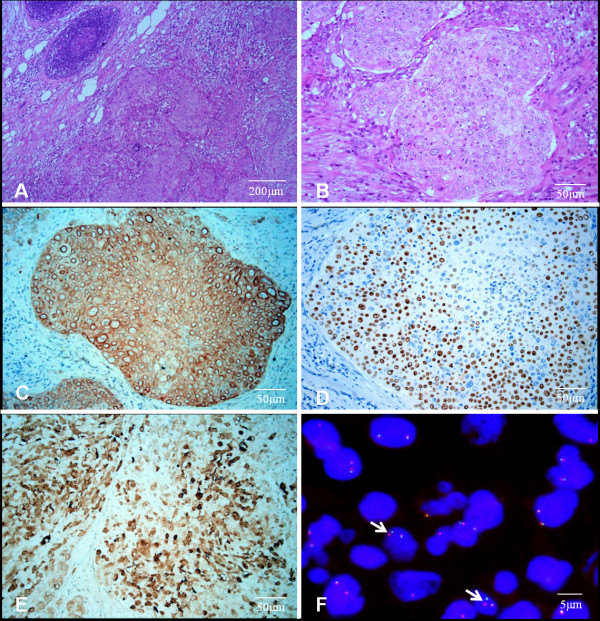
**Right cervical lymph node analysis.** H & E staining **(A and B)**. IHC staining of CK5/6 **(C)**, P63 **(D)**, and ALK (1A4) **(E)**. *ALK* gene translocation (FISH, arrows: split-apart signals for *ALK* gene translocation) **(F)**.

The whole enlarged right cervical lymph node was resected, and followed by biopsy and histologic examination. Hematoxylin and eosin (H & E) staining showed the typical morphology of squamous cell carcinoma cells (Figure 2A and B). Immunohistochemistry (IHC) analysis demonstrated that tumor cells were positive for cytokeratin (CK) 5/6 (Figure 2C) and P63 (Figure 2D), and negative for CK7, CK20, TTF-1, and Napsin A. The positive rate of Ki-67 was 30%. Altogether, these results confirmed the diagnosis of metastatic squamous cell carcinoma. The patient requested treatment with epidermal growth factor receptor (EGFR)-tyrosine kinase inhibitors (TKIs) because dozens of patients with squamous cell carcinoma and EGFR mutations responded well to TKIs at our institute. Amplification refractory mutation system (ARMS) was used to assess EGFR and K-RAS gene profiles to determine the presence of mutated variants. However, evidence of a normal genotype excluded the patient from receiving EGFR-TKI treatment.

As first-line chemotherapy, the patient was initially administered 135 mg/m^2^ (210 mg) of paclitaxel and 80 mg/m^2^ (120 mg) of nedaplatin. During treatment, the patient’s condition did not seem to improve; thus, after a 20-day treatment, a second round of chemotherapy was administered with 75 mg/m^2^ (120 mg) of docetaxel and 80 mg/m^2^ (120 mg) of nedaplatin. Unfortunately, increasing levels of interleukin-6 (IL-6) (25.41 vs 16.03 pg/mL) and carcinoembryonic antigen (CEA) (23.43 vs 7.13 ng/mL) indicated cancer progression, which was confirmed by the presence of multiple metastases in both lungs on CT scan images (Figure 1B). Although not initially indicated, the patient was then administered oral treatment with the EGFR-TKI, erlotinib (1-month trial). However, this treatment showed no efficacy.

After the use of crizotinib was approved by the China Food and Drug Administration (CFDA) in July 2013, the patient underwent *ALK* testing. IHC staining (*Clone 1A4*, *Origene*, 1:200) showed tumor cell positivity for ALK protein (Figure 2E). Then *ALK* break-apart fluorescent in situ hybridization (FISH) was performed on 4-μm, formalin-fixed, paraffin-embedded tissue sections. Slides were hybridized with the LSI *ALK* Break Apart Rearrangement Probe (Vysis, Abbott Molecular, Des Plaines, IL, USA), and read on an epifluorescence microscope (BX41, Olympus, Tokyo, Japan). The lung cancer cell line, NCI-H2228, (American Type Culture Collection-ATCC) was used as positive control. At least 50 tumor cell nuclei were analyzed and at least 15% of interpretable tumor cells harboring break-apart signals were used as the cutoff value [2]. As a result, the presence of *ALK* gene translocation was confirmed (Figure 2F).

The patient underwent crizotinib treatment (250 mg/bid, orally) for 26 days. After this period, symptoms, including neck constriction and cough, were improved. Chest CT scan images demonstrated decrease in tumor size and metastases. According to the Response Evaluation Criteria in Solid Tumors (RECIST) guidelines (version 1.1), such tumor response to crizotinib was classified as partial response (PR) (Figure 1C). Follow-up chest CT scan performed 11 weeks after the beginning of the treatment revealed a dramatic reduction in tumor size and mediastinal lymph node, with nearly no presence of metastases in both lungs (Figure 1D). IL-6 (3.34 vs 25.41 pg/mL) and CEA (1.84 vs 23.43 ng/mL) levels were also reduced 23 weeks after the beginning of the therapy, which demonstrated continuous partial response.

## Discussion

According to the National Comprehensive Cancer Network guidelines, *ALK* testing is not routinely performed in patients with squamous cell lung cancer. Therefore, this patient was not tested for *ALK* until crizotinib was approved for marketing by the CFDA (June 23, 2013). Unfortunately, neither chemotherapy nor EGFR-TKI treatment produced effective tumor response in this patient.

*ALK* gene translocations have been previously detected in patients with lung adenocarcinoma and light or non smoking history [3,4]. To date, two studies have previously reported cases of patients with mixed carcinoma and squamous cell component harboring *ALK* rearrangements [5,6], but these studies did not describe specific therapy or diagnostic procedures. Another report showed that a patient with squamous cell lung cancer, harboring c-MET amplification, had partially responded to crizotinib [7]. Herein, we report the case of a non-smoking woman with squamous cell lung cancer and *ALK* gene rearrangement who experienced remarkable response to crizotinib treatment after failed chemotherapy. In concordance with other studies on patients with lung adenocarcinoma treated with crizotinib [8,9], this patient has remained in remission (PR). Most importantly, such remarkable response was obtained after two courses of failed chemotherapy.

## Conclusion

Despite the low reconstruction rate of *ALK* gene, if applicable, patients with squamous cell lung cancer should have the option of undergoing *ALK* testing and receiving crizotinib treatment. *ALK* testing and subsequent targeted therapy could be an effective option for patients with non-small cell lung cancer who present progression following chemotherapy, radiotherapy, or any other treatment.

### Consent

The patient provided written consent for publication of this case report.

## Abbreviations

EML4: Echinodermmicro tubule associated protein like 4; ALK: Anaplastic lymphoma kinase; NSCLC: Non-small-cell lung cancer; CT: Chest computed tomography; TKIs: Tyrosine kinase inhibitors; EGFR: Epidermal growth factor receptor; IL-6: Interleukin-6; CEA: Carcinoembryonic antigen; CFDA: China Food and Drug Administration; FISH: Fluorescent in situ hybridization; PR: Partial response; IHC: Immunohistochemistry; H & E: Hematoxylin and eosin; ARMS: Amplification refractory mutation system; RECIST: Response Evaluation Criteria in Solid Tumors.

## Competing interests

The authors declare that they have no competing interests.

## Authors’ contributions

QSW carried out the molecular identifications of EGFR and K-ras, and drafted the manuscript. YH provided parts of the clinical treatment data and revised the manuscript. XY participated in the H & E, IHC, and FISH analyses. YBW provided parts of the clinical treatment data. HLX participated in the design and coordination of this study, and helped to revise the manuscript. All authors read and approved the final manuscript.

## Pre-publication history

The pre-publication history for this paper can be accessed here:

http://www.biomedcentral.com/1471-2466/14/83/prepub
